# Supplementary material for: Conserved Small Nucleotidic Elements at the Origin of Concerted piRNA Biogenesis from Genes and lncRNAs
Source: Cells. 2020 Jun 18;9(6):1491. doi: 10.3390/cells9061491 (PMC7349650; doi:10.3390/cells9061491)

SI-MSA2

Supplemental Information: Multiple sequence alignments and consensus sequences of *An. gambiae, Ae. aegypti,* mouse and human snetDNAs

Multiple alignment and consensus of *An. gambiae* snetDNAs matching to 2R_lncs_14 positions 1163-1213:

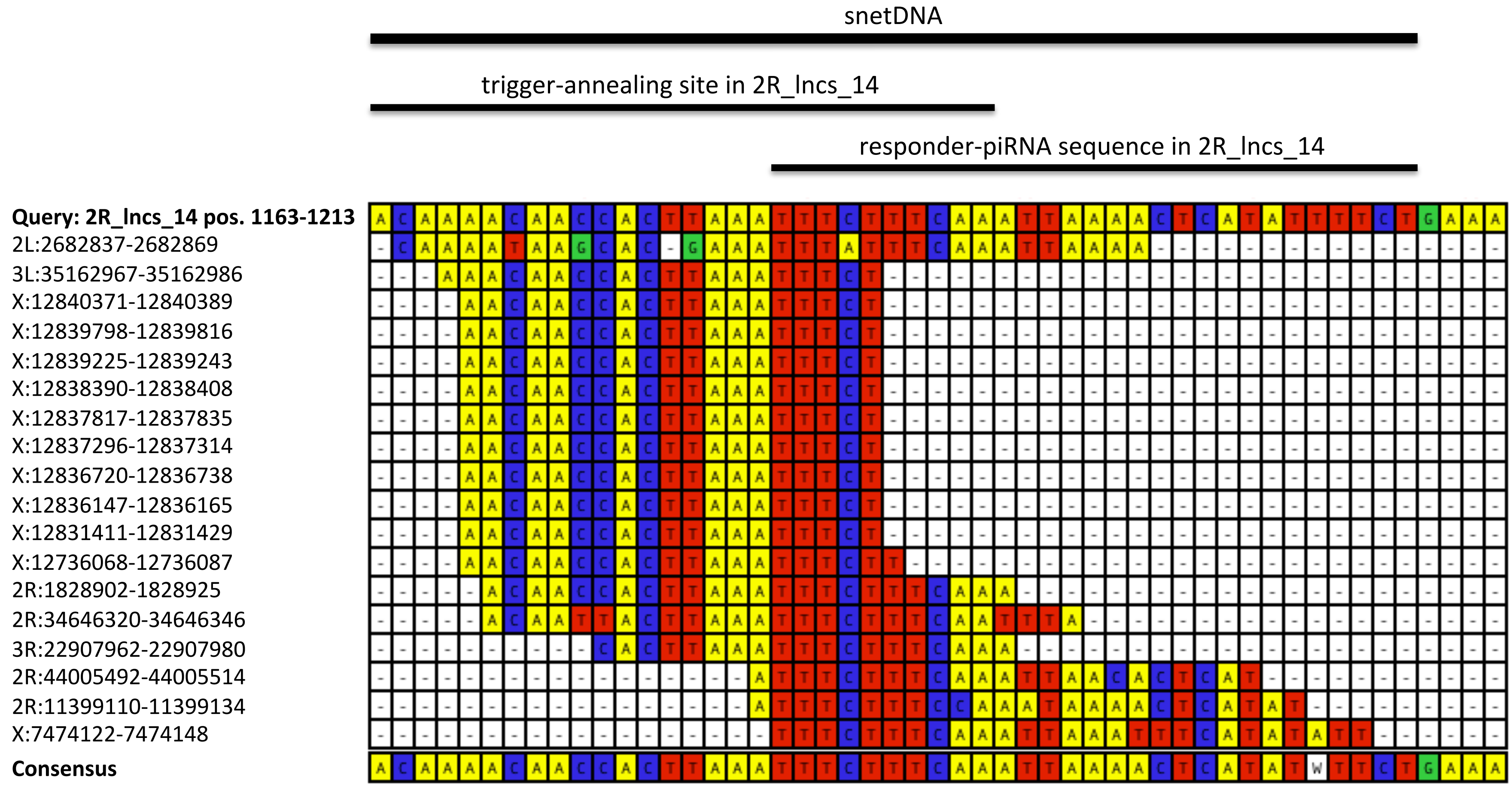

Multiple alignment and consensus of *Ae. aegypti* snetDNAs matching to 2R_lncs_14 positions 1163-1221:

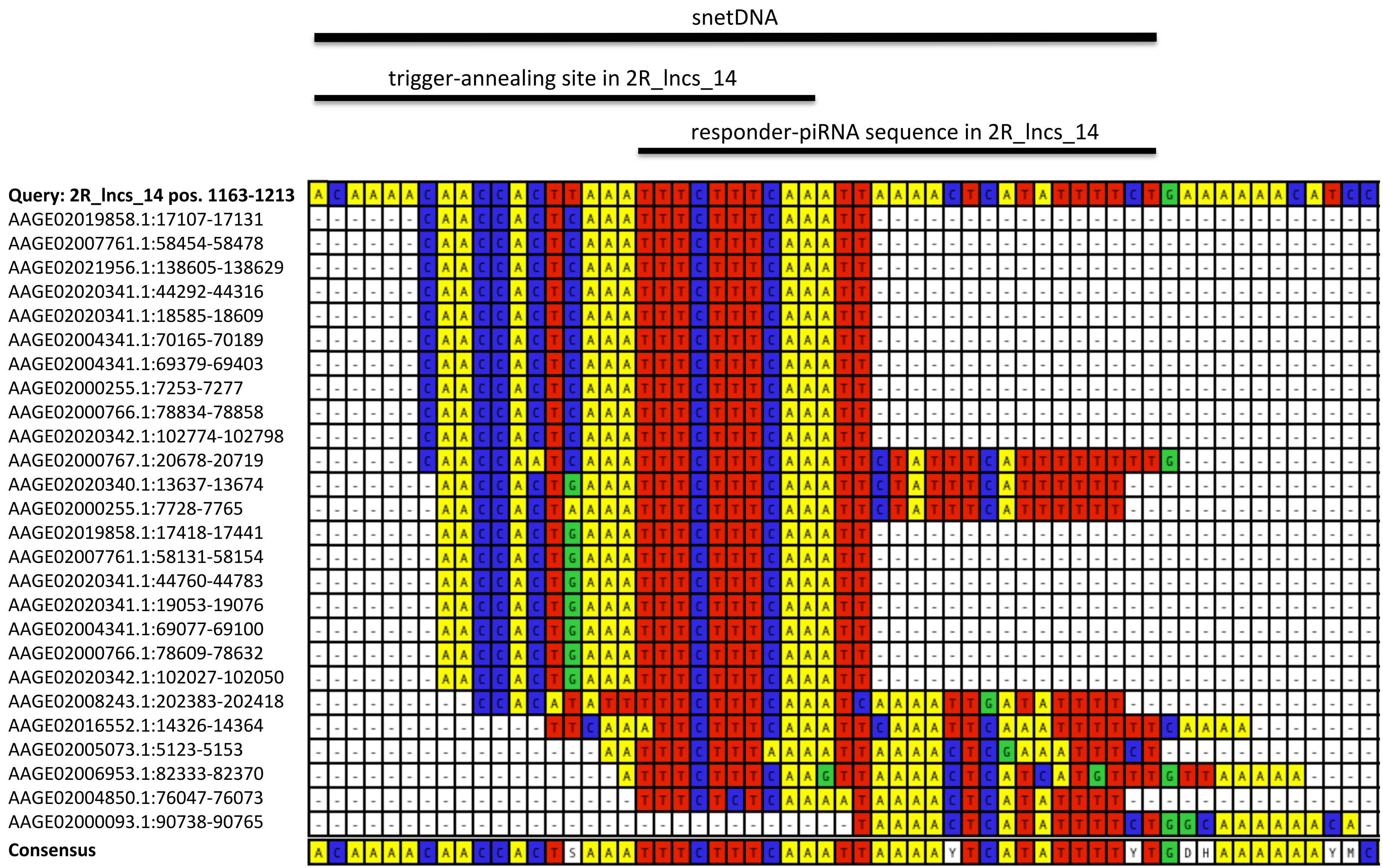

Multiple alignment and consensus of mouse snetDNAs matching to 2R_lncs_14 positions 1163-1231:

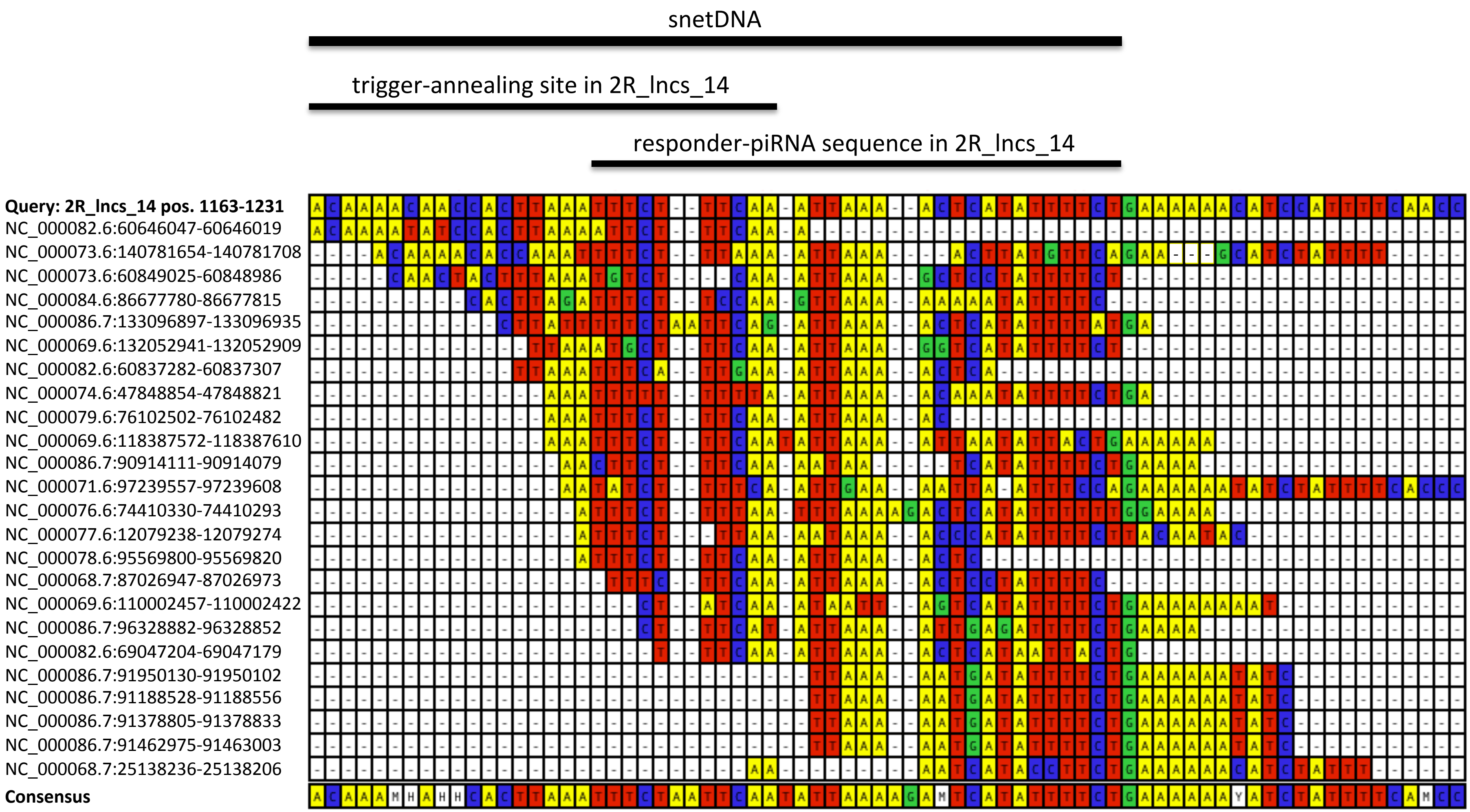

Multiple alignment and consensus of human snetDNAs matching to 2R_lncs_14 positions 1141-1227:

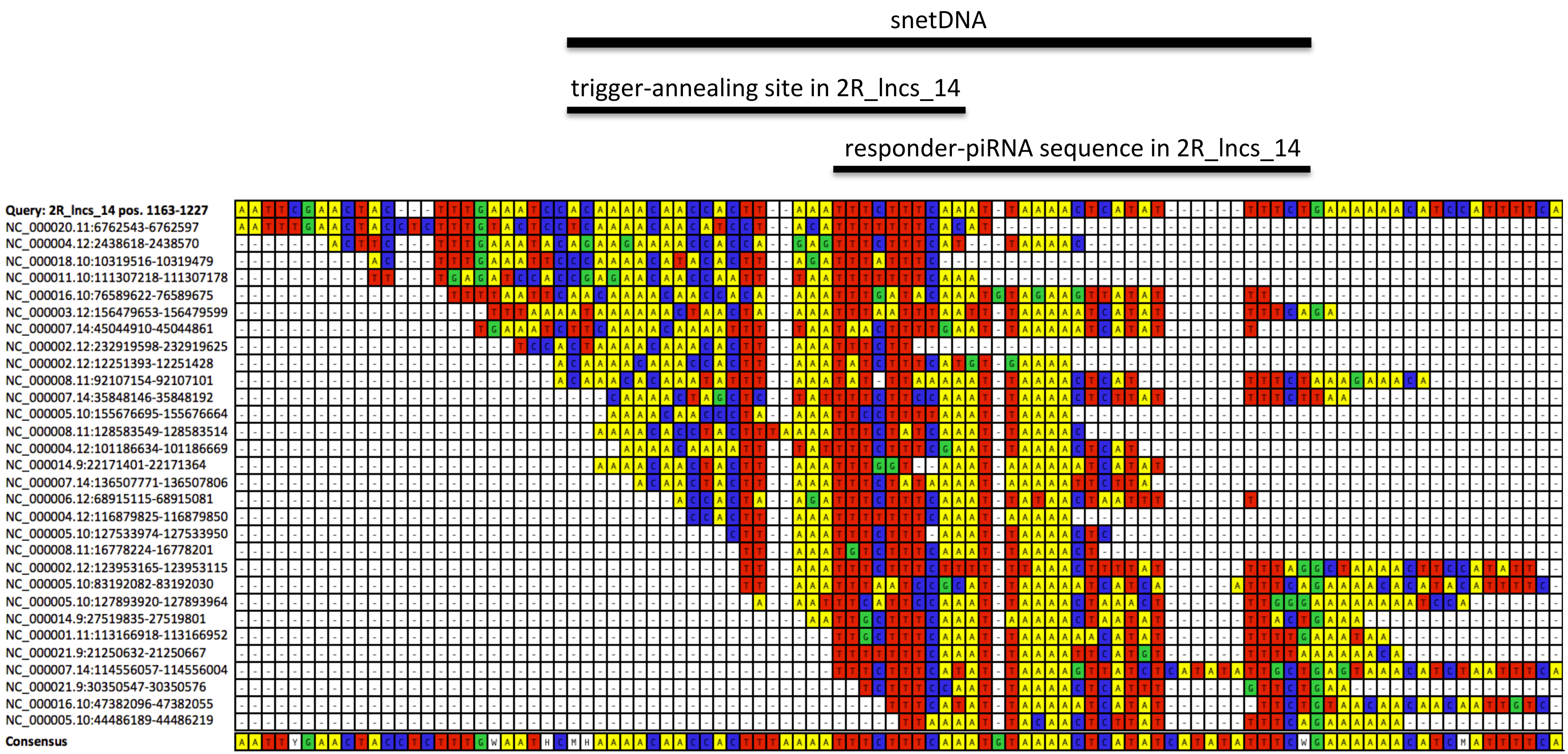

Multiple alignment and consensus of *An. gambiae* snetDNAs matching to AGAP011923-RA positions 231-284:

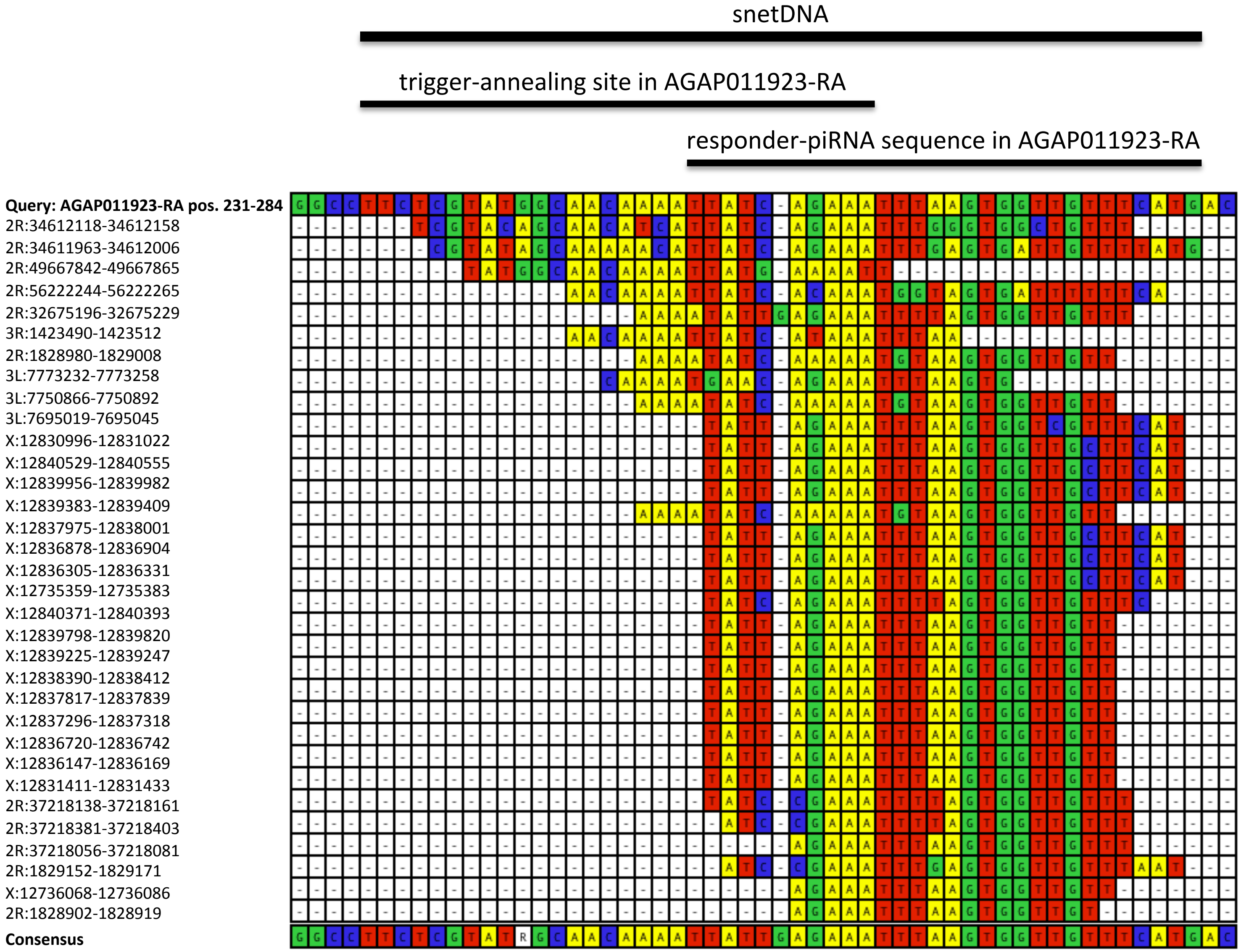

Multiple alignment and consensus of *Ae. aegypti* snetDNAs matching to AGAP011923-RA positions 231-284:

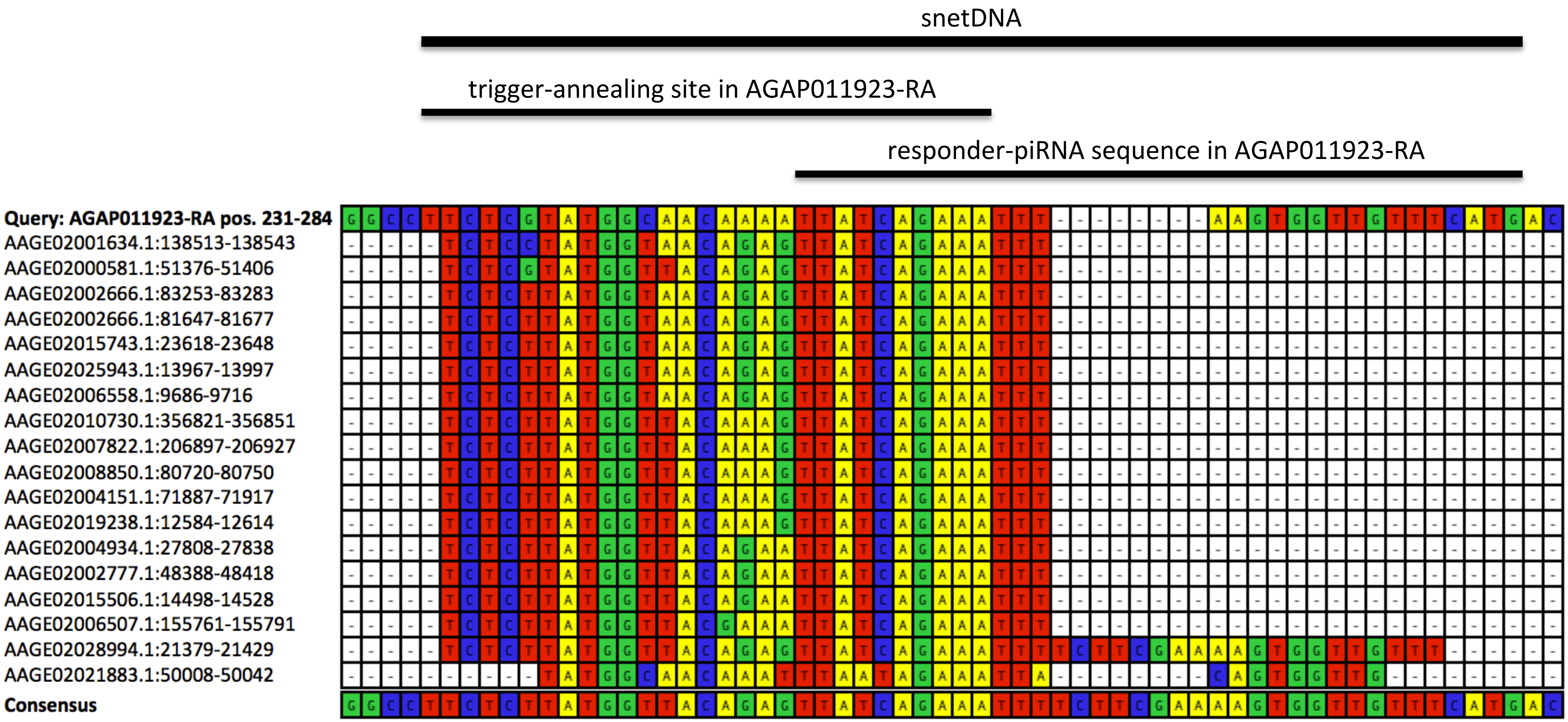

Multiple alignment and consensus of mouse snetDNAs matching to AGAP011923-RA positions 231-284:

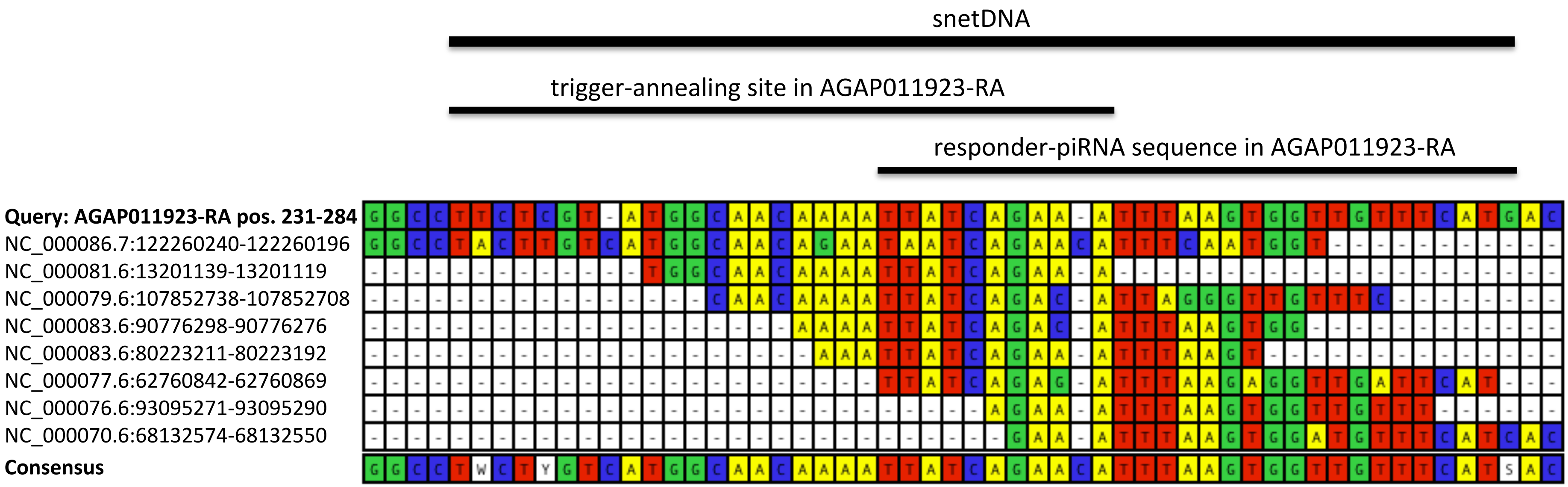

Multiple alignment and consensus of human snetDNAs matching to AGAP011923-RA positions 231-290:

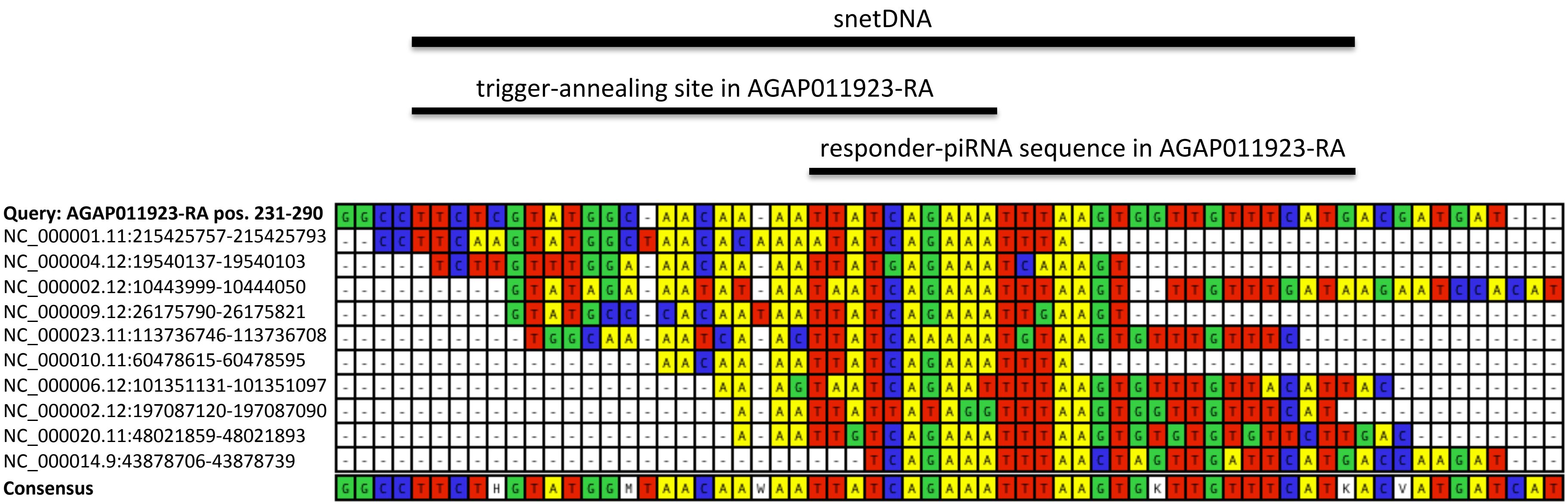

Supplement: Supplementary file 1 [file cells-09-01491-s001.zip › Supplementary-Jensen/Supplementary-3.pdf]
